# Takayasu arteritis: Prevalence and clinical presentation in Switzerland

**DOI:** 10.1371/journal.pone.0250025

**Published:** 2021-06-18

**Authors:** Andrea D. Gloor, Laurève Chollet, Lisa A. Christ, Jennifer L. Cullmann, Harald M. Bonel, Peter M. Villiger

**Affiliations:** 1 Department of Rheumatology, Immunology and Allergology, University Hospital (Inselspital) and, University of Bern, Bern, Switzerland; 2 Department of Diagnostic and Interventional Radiology, University Hospital (Inselspital) and, University of Bern, Bern, Switzerland; Policlinico S. Orsola-Malpighi, ITALY

## Abstract

**Objective:**

Takayasu arteritis (TAK) is a rare immune-mediated vasculitis of the aorta and its branches. Aims were to calculate prevalence and incidence in Switzerland, to assess disease activity and performance of MR-Angiography (MRA).

**Methods:**

31 patients were recorded in a database, 27 were followed prospectively up to 3 years. Prevalence was calculated based on data of the national statistical bureau. Disease activity was defined using the revised EULAR criteria. MRA depicted stenotic changes and aortic wall enhancement.

**Results:**

A disease prevalence of 14.5/1.000.000 inhabitants and an incidence of 0.3/1.000.000 per year was calculated. Aortic wall enhancement was found in 10 patients while in clinical and serological remission. EULAR criteria missed 5 patients with disease activity with isolated elevations of ESR/CRP. Arterial stenosis did not change over time in 5 cases, it improved in 2 and increased in 7. At follow-up 16 patients were treated with tocilizumab, 11/16 in monotherapy, 5 patients were treatment-free, 25/27 stayed in remission.

**Conclusion:**

In addition to prevalence and incidence, our data show that MRA qualifies to detect subclinical disease activity, but, on the other hand, that EULAR criteria may miss disease activity in case of isolated elevation of ESR/CRP.

## Introduction

Takayasu arteritis (TAK) is a rare immune-mediated large vessel vasculitis (LVV) of granulomatous nature that mostly affects women of childbearing age [1]. The reported prevalence varies between 4.7-33/1’000’000 in Western European countries and 30-40/1’000’000 in Asian populations [2–7]. TAK predominantly affects the aorta and its major branches, it may involve the aortic valve and coronary arteries. Clinical signs and symptoms of active disease are mild and non-specific; systemic inflammation is typically low to moderate [3, 4]. The smoldering inflammation may result in fibrosis and destruction of the vessel wall, eventually leading to stenosis and/or formation of aneurysms. Based on the insidious onset, diagnosis is often delayed by many years, and treatment starts at a time of established disease damage. Accordingly, the Chapel Hill Classification Criteria are largely based on disease damage [8, 9]. The revised EULAR recommendations for the management of LVV states that in addition to conventional immune suppressive agents the biologics infliximab and tocilizumab may be prescribed in TAK with comparable efficacy [10].

Recommendations regarding imaging of LVV have recently been published by EULAR [11]. Based on its quality to assess vessel wall inflammation as well as vessel anatomy, but also to avoid radiation exposure in young females, magnetic resonance angiography (MRA) qualifies best to confirm diagnosis and monitor evolution of disease.

The objectives of this study were to calculate the disease prevalence and the incidence in Switzerland, to describe clinical features of TAK patients and to assess performance of MRA and EULAR criteria in this cohort.

## Methods

### Patients and data collection

In this single center observational cohort study performed in the Department of Rheumatology, Immunology and Allergology at the University Hospital of Bern, the data of clinical, laboratory and imaging findings of 31 patients were collected. Structured interviews, clinical examination and blood collection were performed at the time of inclusion in all patients and during follow-up (2016–2019) in 23 patients. Four patients were followed up by a telephone interview, 3 were enrolled very recently, therefore no follow-up has taken place yet and one patient was lost during follow-up. In all patients, imaging was performed as described below. Thirty out of 31 patients fulfilled the 1990 American College of Rheumatology (ACR) criteria for TAK. In brief, the ACR criteria are met and the diagnosis of TAK shall be made if at least 3 of the following 6 criteria are present: Age at disease onset <40 years, claudication of extremities, decreased brachial artery pulse, blood pressure difference >10 mmHg of the arms, bruit over subclavian arteries or Aorta and imaging abnormalities of the aorta and/or large arteries [12]. All patients were aged over 18 years. The data were entered in a data base (secuTrial ®) developed and run by the Clinical Trial Unit (CTU) of the University of Bern. The study was approved by the local ethics committee (Kantonale Ethikkommission Bern; KEK, KEK-BE: 2016–00338) and performed in accordance with the Declaration of Helsinki. All patients gave written informed consent.

### Criteria for disease activity

The revised EULAR criteria were used to asses disease activity [10]. In addition to key symptoms and key findings* (see below), at least one of the following criteria must be present: a) current activity on imaging or biopsy, b) ischemic complications attributed to large vessel vasculitis, c) persistently elevated inflammatory markers (after exclusion of other causes). To quantify elevated inflammatory markers, the C reactive protein (CRP) (standard test) and the erythrocyte sedimentation rate (ESR) were assessed in the peripheral blood.

*Typical signs or symptoms suggestive of active Takayasu arteritis (EULAR recommendation)

1. Key symptoms
  - New onset or worsening of limb Claudication
  - Constitutional symptoms (eg, weight loss >2 kg, low-grade fever, fatigue, night sweats)
  - Myalgia, arthralgia, arthritis
  - Severe abdominal pain
  - Stroke, seizures (non-hypertensive), syncope, dizziness
  - Paresis of extremities
  - Myocardial infarct, angina
  - Acute visual symptoms such as amaurosis fugax or diplopia
2. Key findings on clinical examination
  - Hypertension (>140/90 mm Hg)
  - New loss of pulses, pulse inequality
  - Bruits
  - Carotidynia

### Vessel involvement assessed by imaging modalities

MRA, doppler ultrasonography, computed tomography angiography (CTA) or positron emission tomography–computed tomography (PET-CT) were used for diagnosis. MRA or CTA was performed in all patients at least once and in 24 patients as a follow-up. These were analyzed by two vasculitis-experienced radiologists (J.C. and H.B.), who were blinded to all clinical and laboratory data. In addition to assessment of stenosis and aneurysms of the large arteries, vessel wall signals of the aorta were judged in 28 patients according to a published protocol [13]. The first and last MRA or CTA were analyzed in 22 patients regarding anatomical changes and in 18 regarding vessel wall signals. In two patients, a different imaging modality was used at the initial examination and at the follow-up. Therefore, they were excluded from the analysis of the anatomical changes. Arterial distribution was classified using the angiographic classification of the 1994 International TAK Conference based on the distribution of the involved vessels in MRA as follows [14]:

- Type I; branches of aortic arch
- Type IIa; ascending aorta, aortic arch with its branches
- Type IIb; ascending aorta, aortic arch with its branches and thoracic descending aorta
- Type III; thoracic descending aorta, abdominal aorta and/or renal arteries
- Type IV; only abdominal aorta and/or renal arteries
- Type V; combination of Type IIa and Type IV

### Prevalence and incidence

For calculation of prevalence, an anonymized list of all patients fulfilling ACR criteria was used (including 5 patients who refused to have their data collected and excluding the patient not fulfilling the ACR criteria). Based on the postal code, municipality was identified. Population density of each municipality/canton was extracted from tables of the Bundesamt für Statistik (the Swiss Federal Office of Statistics) [15]. Boundaries of each municipality/canton were retrieved from the Bundesamt für Landestopografie swisstopo [16]. The spatial distribution of patients was visualized on a map. The prevalence was calculated at the end of the follow-up; there were no deaths of TAK patients recorded during the study and the follow-up. The catchment area of the University Hospital is the canton of Bern and areas of neighboring German speaking cantons adding up to 1.5–2 millions of inhabitants.

### Statistical analysis

Data were exported from secuTrial®. Statistical analysis was performed using IBM SPSS Statistics 25 and GraphPad Prism 8.0 software. Continuous and categorical variables are presented as median [lower quartile, upper quartile] or number and percentage of patients. In case of missing data (n<31), the resulting number of data is added and the %-age refers to this number.

## Results

### Patient population

31 patients aged between 18–83 years were enrolled in the study. All except one fulfilled the ACR TAK criteria [12]. This one patient had 2 (age at disease onset ≤ 40 years and abnormalities in imagine) out of the 6 ACR criteria. Based on the phenotype of the disease (chronically increased inflammatory values, asthenia and malaise) as well as exclusion of differential diagnoses, the diagnosis of TAK was retained. The TAK diagnoses were made between 1971 and 2019. Baseline demographic patient’s characteristics are summarized in Table 1. Eight out of 31 patients (26%) presented first symptoms before the age of 20 years and six (19%) were 40 years or older at disease onset. Five of these patients (16%) were between 40 and 45 years old and one was 52 when the first signs were documented. Importantly, all patients aged more than 40 years showed pronounced vascular changes, suggesting a prolonged subclinical disease process. Thus, in all these patients, the TAK presumably began before the age of 40. The median diagnostic delay was 6 months. Nine patients (29%) were diagnosed more than 5 years after the onset of first symptoms. S1 Fig shows the disease duration of all patients.

**Table 1:** Demographic profiles (n = 31).

	Min.		Max.	Median (Quartiles)
Age at clinical onset (years)	2		52	27 (18, 36)
Age at time of diagnosis (years)	14		62	28 (23, 38)
Age at time of inclusion (years)	18		83	37 (30, 53)
Delay until diagnosis (months)	0		204 (17 years)	6 (1, 96)
	**n = 31**	**(%)**		
Female sex	30	(97)		
Ethnic origin Swiss	21	(68)		
Ethnic origin non-Swiss (total)	10	(32)		
• Asian	5			
• Middle east	2			
• South America	2			
• Serbia	1			

Values represent median (lower quartile, upper quartile, and minimum to maximum), and number of patients with percentage (%).

### Prevalence and incidence in Switzerland

**Fig 1:**
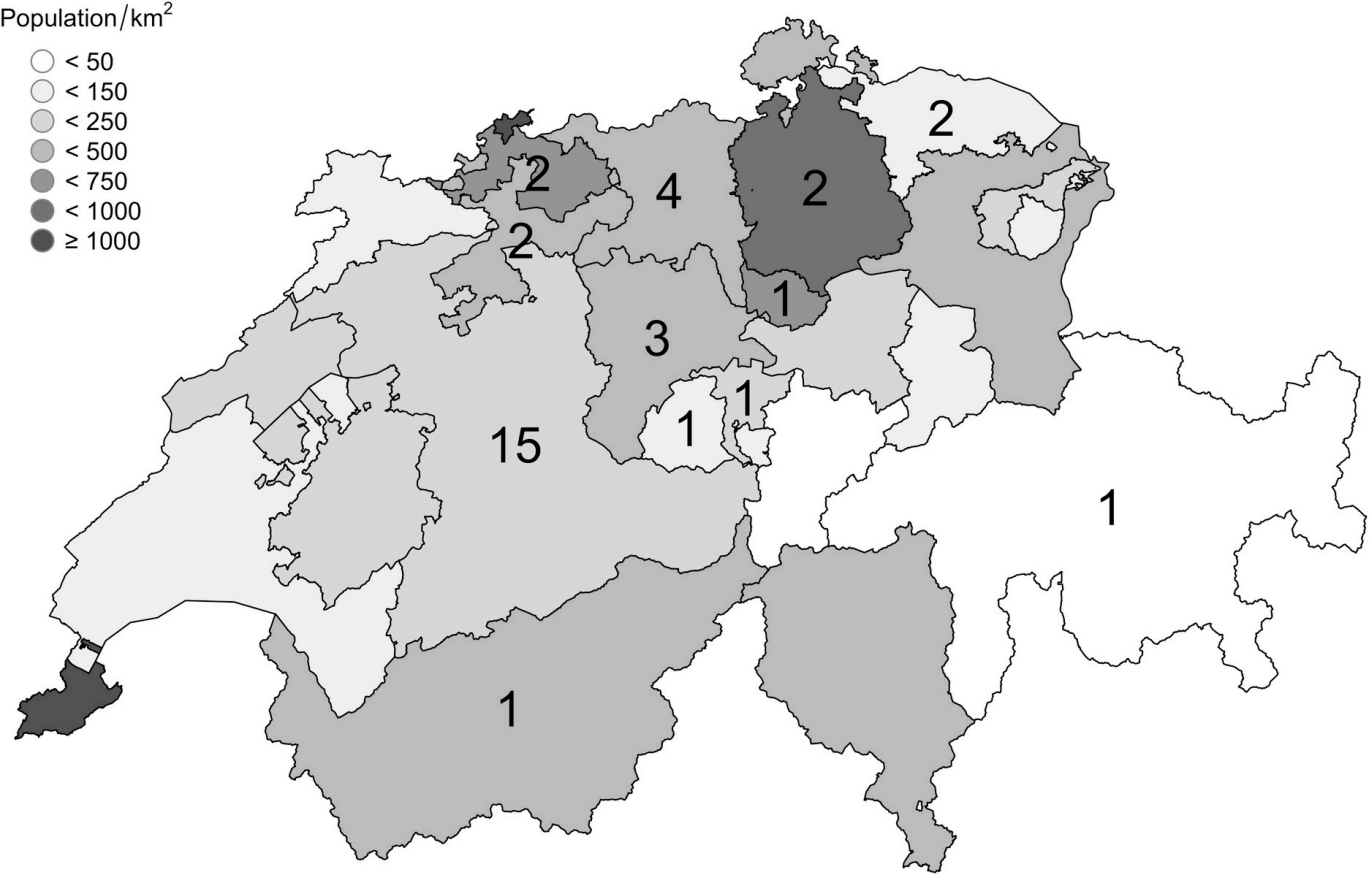
TAK patients in Switzerland. Displayed are Swiss cantons and their populations per km^2^. The number of patients (in black) are displayed per canton.

Five patients refused to have their data recorded and one patient did not fulfil ACR criteria [12]. Thus, calculation of prevalence was based on 35 patients and performed in anonymized form. As displayed in Table 2 and Fig 1, fifteen patients lived in the canton of Bern and 20 in other cantons.

**Table 2:** Prevalence in Switzerland (n = 35).

	Canton Bern	Switzerland
Residents [14]	1.034.977	8.544.527
• <40 years	458.503	3.977.387
• ≥ 40 years	576.474	4.567.140
Patients	15	35
• <40 years	4	19
• ≥ 40 years	11	16
Prevalence	14.5/1.000.0005	
• <40 years	8.7/1.000.000	
• ≥ 40 years	19.1/1.000.000	

Values represent number of residents and patients and prevalence expressed as cases per million residents.

The prevalence in the canton of Bern is 14.5/1.000.000 inhabitants. The prevalence of patients <40 years is 8.7/1.000.000 while the prevalence of patients 40 years or older is 19.1/1.000.000. Over a period of 50 years, 15 patients were diagnosed in the Canton of Bern. Based on a population of 1.034.977, the incidence is therefore 0.3/1.000.000/year.

### Clinical features and laboratory findings at disease onset and at inclusion in the registry

The most frequent signs and symptoms at disease onset and at time of inclusion are presented in Table 3. Two thirds of patients initially suffered from general symptoms such as asthenia, elevated temperatures, weight loss and night sweat. General symptoms where less frequent at time of inclusion (26%). Elevated acute-phase reactants such as C-reactive protein (CRP) and/or erythrocyte sedimentation rate (ESR) were found in 85% of the patients at disease onset, whereas only in 19% at time of inclusion. In 26/28 patients (84%) at least one of the following 3 clinical findings was present at disease onset: bruits over big arteries, diminished or absent pulses, blood pressure difference > 10mmHg between the right and the left arm. In two patients (6%) blood pressure was not measurable at all. Three patients (10%) did not suffer from signs of vascular involvement or general symptoms before or at the time of diagnosis. In these cases, the arteritis was diagnosed based on bruits observed in physical examination, unexplained elevated CRP levels and aortitis as an incidental finding in an MR-enteroclysis, respectively.

**Table 3:** Clinical features at disease onset and at inclusion.

	disease onset		inclusion	
Symptoms	n = 31	(%)	n = 31	(%)
Asthenia/ fatigue/ feeling sick	20	(65)	8	(26)
Claudication of upper limbs	17	(55)	19	(61)
Carotidynia and sore throat	17	(55)	4	(13)
Dizziness or subclavian steel phenomena	10	(32)	7	(23)
Weight loss	9	(29)	0	(0)
Claudication of lower limbs	7	(23)	4	(13)
Muscle tension head and neck region	7	(23)	9	(29)
Arthralgia	6	(19)	9	(29)
Cold hands	6	(19)	7	(23)
Night sweat	4	(13)	3	(10)
Exertional dyspnoea	4	(13)	3	(10)
Headache	4	(13)	1	(3)
Syncope	4	(13)	1	(3)
Visual disturbance	3	(10)	2	(6)
Incidental finding	3	(10)	0	(0)
Cold feet	2	(6)	7	(23)
Myalgia	2	(6)	1	(6)
Nausea, Vomiting, systemic inflammation	1	(3)	0	(0)
Retrosternal chest pain	1	(3)	1	(3)
Palpitation or tachykardia	0	(0)	8	(26)
Abdominal pain	0	(0)	1	(3)
Hemoptysis	0	(0)	1	(3)
**Findings**	n = 31*		n = 31*	
Elevated acute-phase reactants (CRP, ESR)	22/26	(85)	6	(19)
Bruits over the big arteries	19/25	(76)	17	(55)
Diminished/ absent pulse	16/25	(64)	19	(61)
Blood pressure difference (left/right) >10mmHg	15/25	(60)	17/29	(55)
Fever/ elevated temperatures	15	(48)	1	(3)
Arterial Hypertension	6	(19)	16	(48)
Hepatomegaly	3	(10)	0	(0)
Blood pressure not measurable	2/25	(4)	3	(10)
Pleural effusion	1	(3)	0	(0)
Myocarditis	1	(3)	0	(0)

C-reactive protein (CRP), erythrocyte sedimentation rate (ESR).

*n = 31 if not specified. Values represent number of patients with percentage (%).

### Disease activity at inclusion and at follow-up

At time of inclusion, 5 patients (16%) showed signs and symptoms of disease activity, the remaining 26 (84%) were in remission. One of the five patients subsequently achieved remission which persisted even after discontinuation of immunosuppressive therapy. One patient achieved remission but relapsed once during the follow-up period of 3 years; one did not achieve complete remission (≥3 months) during a follow-up time of 10 months despite immunosuppressive treatment.

**Fig 2:**
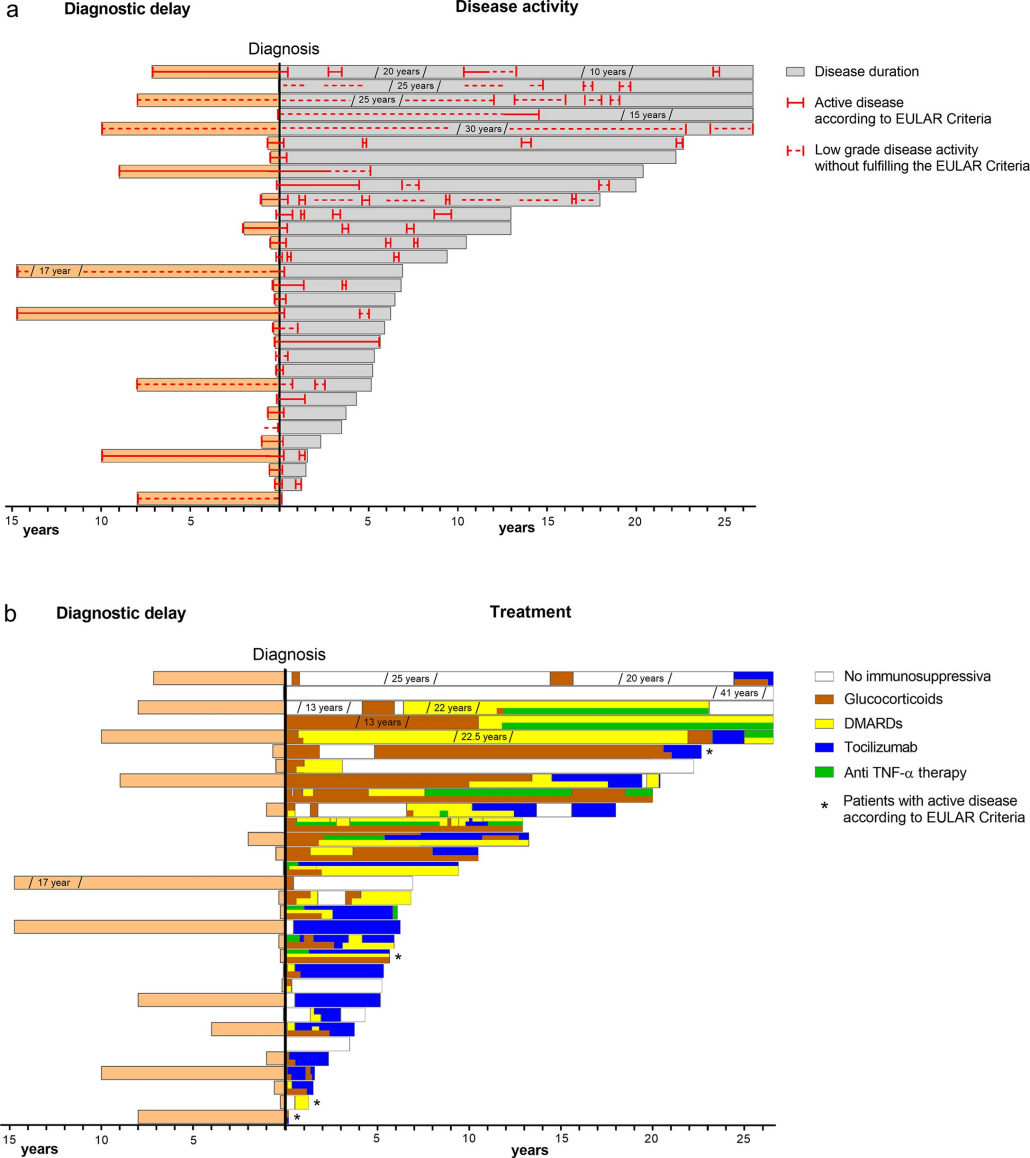
Disease activity and medication. Disease activity (A) and medication of each patient over time (years) (B). 27/31 patients were followed prospectively up to 3 years. DMARDs: Disease-modifying antirheumatic drugs.

Six patients (19%) suffered one relapse over time. Five patients (16%) showed increased signs of inflammation (CRP and/or ESR) over several weeks during follow-up without clinical signs or symptoms and had, therefore, no active disease according to EULAR criteria. Fig 2A illustrates the disease activity during the disease course of each patient.

#### Vessel involvement at inclusion and at follow-up

**Fig 3:**
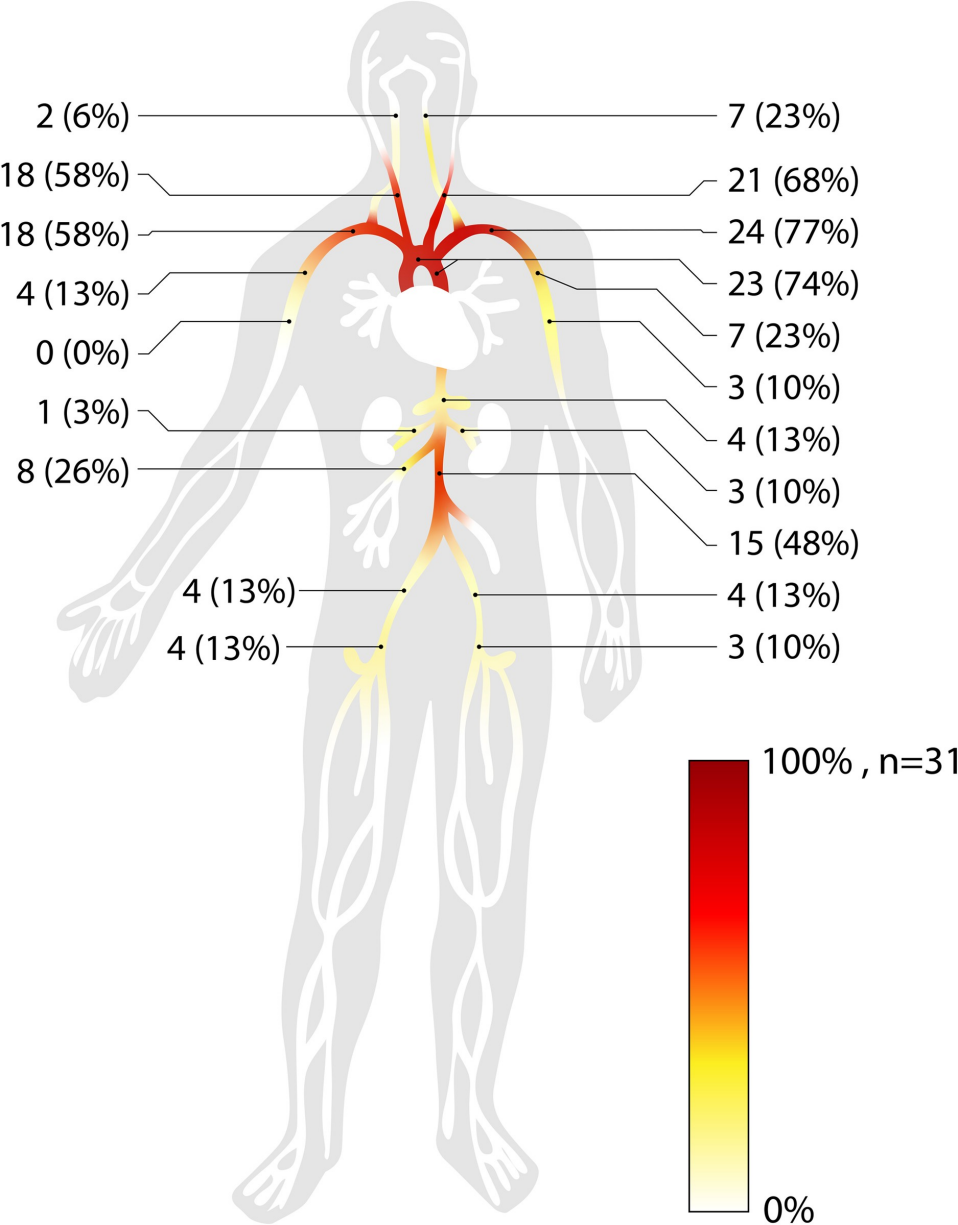
Vessel involvement. Pattern of vessel involvement of 31 TAK patients as determined by imaging and/or clinical findings.

Vascular involvement is presented in Fig 3. The most affected vessel was the left subclavian artery, followed by the aortic arch and the thoracic aorta, the left and the right common carotid artery and the right subclavian or innominate artery. Of 14 patients arterial stenosis did not change in 5 cases (median observation time: 62 months), it improved in 2 (median observation time: 144 months) and increased in 7 (median observation time: 58 months). Nine patients (29%) underwent interventional revascularization and/or received an arterial bypass or a vascular graft, which limited the possibility of assessing the course of these stenosis. As displayed in Table 4, Typ V (52%) arteritis was the most common pattern according to the 1994 angiographic classification for TAK [14].

**Table 4:** Angiographic type (n = 31).

Type	n.	(%)
I	5	16
IIA	2	6
IIB	8	26
III	0	0
IV	0	0
V	16	52

Values represent number of patients with percentage (%).

### Disease activity assessed by MRA

Based on a score ≥ 2 in MRA, 16/28 patients (57%) showed inflammation in the vessel wall. Of these 16, 10 patients (63%) were in clinical and humoral (CRP/ESR) remission, while 6 patients (37%) showed clinical and/or humoral signs of disease activity at the time of MRA. Of 12 patients with an activity score <2, 9 (75%) demonstrated no clinical or humoral signs of disease activity, while 3 patients (25%) had clinical and/or humoral signs of active disease.

Follow up assessment of the vessel wall signal was performed in 18 patients. Six out of these 18 patients (33%) showed a decrease (median observation time: 96 months), while 10/18 patients (56%) had stable signal intensity (median observation time: 61 months). Two patients (11%) showed a fluctuating signal intensity (observation time: 177 and 46 months, respectively).

### Medication

Fig 2B describes in detail the chronology of the medication of all patients. At time of inclusion 24 patients (77%) received immunosuppressive therapy (drugs listed in S1 Table), as monotherapy in 12/24 (50%). Seven patients (23%) did not receive any immunosuppressive agents at study inclusion. One patient started an immunosuppressive therapy during follow-up, one patient was lost during follow-up and the last one was enrolled recently.

GC were used in 25 patients (80%). In 20/24 (83%), GC could be tapered and stopped and 17/24 (71%) remained GC free for at least 3 years, one patient was not followed. Six patients (19%) achieved remissions with DMARDs or biological agents or without therapy.

At the end of follow-up, 5 patients (16%) were without medication and showed no signs of active disease. Twenty-one out of 31 patients (68%) were on biologicals, either tocilizumab (n = 16, 52%) or anti TNF-α therapy (infliximab; n = 4, 19%, certolizumab: n = 1, 5%). Eleven out of the 16 patients (69%) were on tocilizumab monotherapy, of these were 10 (91%) in remission.

## Discussion

This is the first study reporting the prevalence and the incidence of TAK in Switzerland, describing disease characteristics as well as assessing performance of MRA and of recently proposed EULAR disease activity criteria.

Data of the canton of Bern, which lies within the catchment area of the University Hospital and represents a seventh of the whole population of Switzerland, was used to calculate prevalence. With 14.5/1.000.000 inhabitants, the prevalence is 3-fold higher as compared to a prior reported prevalence of 4.7/1’000’000 in Western European countries; it is similar to recent data of Sweden and Norway [3, 6, 7]. The prevalence of the Scandinavian countries may be higher than those of other European countries, as cases were extracted out of established hospital registries. As diagnosis of TAK is straightforward and all but one patient of our cohort fulfilled classification criteria, it is unlikely that we recorded false positives. On the other hand, the non-specific and often very mild symptoms suggest a substantial number of non- or not-yet diagnosed cases. This is corroborated by the fact that three out of 31 patients were incidentally diagnosed. Collectively, the effective prevalence might be higher than the calculated. The characteristics of the canton of Bern regarding geography, localisation and population allows extrapolation to the whole country.

In contrast to Giant Cell Arteritis, TAK classification is based on stenotic changes of arteries leading to reduced pulse, side differences in blood pressure, claudication and bruits on auscultation [1, 17]. In accordance, at time of diagnosis, more than half of our patients suffered from reduced or absent pulse and arm claudication, preferentially the left arm. And in line, suspicion of TAK could have been raised in more than 90% of our patients by auscultation of large thoracic arteries, comparison of the blood pressure of the arms or palpation of the radial artery.

A question arises in this context: Do the classification features represent active vessel wall inflammation or established disease damage. We tried to answer it with our imaging data. MRA provides information about stenotic/aneurysmatic arteries but also about active vessel wall inflammation [18]. In our Cohort, the follow-up examinations showed that stenotic arteries remained unchanged or slowly worsened despite lasting clinical and serologic remission. This implicates that arterial stenosis at diagnosis most often represent established disease damage. On the other hand, MRA showed vessel wall enhancement in more than half of the patients. Remarkably, almost 2/3 of these patients were in clinical and serological remission. This indicates that MRA may detect subclinical disease activity and that it adds information to conventional laboratory measures and clinical signs and symptoms. In summary, MRA not only provides angiographic information, but it may help to detect subclinical local disease activity and thereby guide treatment intensity.

Compared to the ultrasound technology, which has become the method of choice to diagnose giant cell arteritis, MRA has a higher standardisation of data acquisition and bears the possibility to investigate multiple cranial and extracranial arteries including the aorta at the same time, which might reduce the probability of missing inflammation in case of skip lesions. The limitations of MRA are a restricted availability, costs and possible adverse effects of contrast agents. PET-CT as the third established method is expensive, has an even more limited availability and is based on radiation. PET-CT, therefore, does not qualify for repetitive assessment of primarily female patients in reproductive age [18, 19].

While disease activity was present in over 80% of patients at diagnosis, the percentage had dropped at time of study inclusion to 16%, as assessed using EULAR criteria [10]. This decrease is mainly due to treatment initiation with glucocorticoids and DMARDs. EULAR criteria defining active disease ask for more than one sign and/or symptom. In our cohort, however, we observed isolated elevation of CRP/ESR in 5 patients. As of the lack of another explanation, TAK remains as cause of this finding. The high percentage of patients with isolated elevation of acute phase reactants questions the reliability of EULAR criteria and strongly suggest subclinical disease activity. The problem of missing smoldering disease becomes even more important, if biologic agents are prescribed, which block the IL-6 pathway, and thereby blunt the acute phase reaction [20].

GC were prescribed to induce remission and to control relapse in most cases. Fortunately, they could be tapered and stopped in all but four cases. At follow-up, more than 2/3 of patients were off-steroids for more than three years. Thus, GCs were the treatment of choice to induce remission and control relapse, as proposed in the revised EULAR recommendation for management of LVV [10]. As displayed in Fig 2, our data show a gradual shift from GC plus DMARDs to biologic agents in more recently diagnosed cases, in a first step to infliximab and in a second to tocilizumab. At follow-up, twenty-two patients were treated with biologic agents, whereof half with tocilizumab in monotherapy. Thus, in line with EULAR guidelines [10], our data argue for the prescription of anti-TNF agents or tocilizumab. However, as cautioned above, tocilizumab suppresses production of acute phase reactants, rendering monitoring of disease activity more difficult [20]. Collectively, our data suggest that in these cases, vessel wall enhancement in MRA may be helpful to assess disease activity.

The major limitations of our study are the low sample size, which does not allow statistical calculations, its partly retrospective nature, the inhomogeneous follow-up duration and the fact that it is a single center study. On the other hand, the sample size allowed a meticulous analysis of the patient charts, and the integration of the data in a registry provided a standardized follow-up.

## Conclusions

In summary, the study reports the prevalence and incidence of TAK in Switzerland. The MRA data document established disease damage at diagnosis in the majority of cases, and they show subclinical disease activity in one third of patients. Thus, MRA adds important information and may thereby help to guide treatment. In Our Cohort EULAR criteria for active disease missed those patients in clinical remission with isolated elevation of acute phase reactants (ESR/CRP). Follow-up data strongly argue for prescription of the biologic agents tocilizumab and infliximab. Tocilizumab blunts the acute phase response [20]. Thus, an anti-IL-6 strategy may pose a problem in detecting disease activity. In such situations, MRA may provide helpful information about local vessel wall inflammation.

## Supporting information

S1 Fig Disease duration of 31 TAK patients. 14/31 patients are residents of the Canton of Bern (marked dark grey).
(DOCX)

S1 Table Treatment at inclusion, at the end of follow-up and during course of disease. (DOCX)
