# Supplementary material for: Takayasu arteritis: Prevalence and clinical presentation in Switzerland
Source: PLoS One. 2021 Jun 18;16(6):e0250025. doi: 10.1371/journal.pone.0250025 (PMC8213155; doi:10.1371/journal.pone.0250025)
Supplement: S1 Fig — 14/31 patients are residents of the Canton of Bern (marked dark grey). (DOCX) [file pone.0250025.s001.docx]

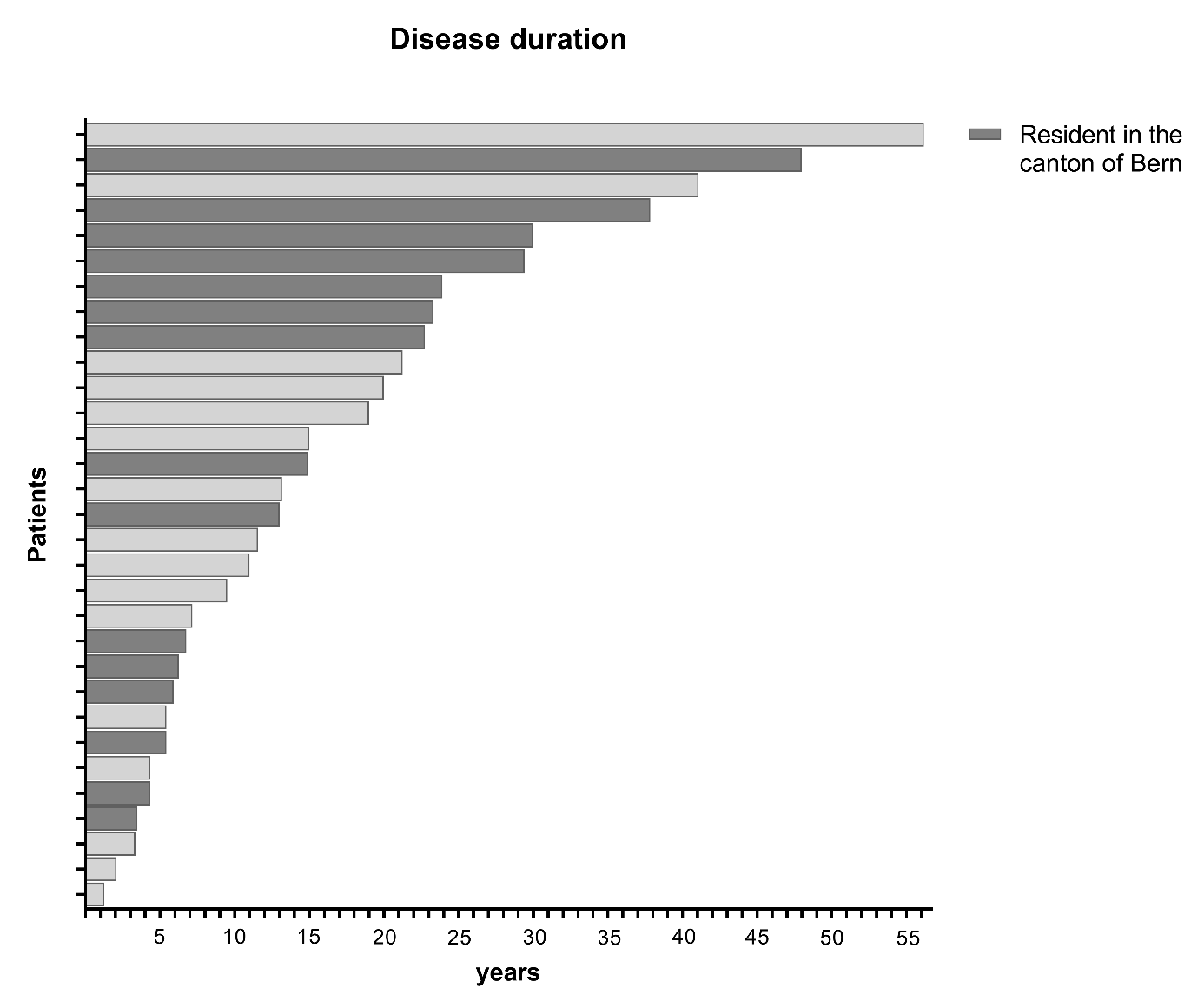


**S1 Fig. Disease duration of 31 TAK patients.** 14/31 patients are residents of the Canton of Bern (marked dark grey).
