# Supplementary material for: Takayasu arteritis: Prevalence and clinical presentation in Switzerland
Source: PLoS One. 2021 Jun 18;16(6):e0250025. doi: 10.1371/journal.pone.0250025 (PMC8213155; doi:10.1371/journal.pone.0250025)
Supplement: S1 Table — (DOCX) [file pone.0250025.s002.docx]

| **S1 Table.**  **Treatment at inclusion (n=24/31)** | | |
| --- | --- | --- |
| **Drug** | **n.** | **(%)** |
| Tocilizumab | 15 | (45) |
| Prednisone | 13 | (42) |
| Methotrexate | 7 | (23) |
| Infliximab | 2 | (6) |
| Azathioprine | 2 | (6) |
| Cyclosporine | 1 | (3) |

| **Treatment at the end of follow-up (n=22/27)** | | |
| --- | --- | --- |
| **Drug** | **n.** | **(%)** |
| Tocilizumab | 16 | (59) |
| Methotrexate | 5 | (19) |
| Prednisone | 4 | (15) |
| Infliximab | 4 | (15) |
| Azathioprine | 3 | (11) |
| Cyclosporine | 1 | (4) |
| Certolizumab | 1 | (4) |

| **Treatment during course of disease (n=29/31)** | | |
| --- | --- | --- |
| **Drug** | **n.** | **(%)** |
| Prednisolone | 25 | (81) |
| Tocilizumab | 21 | (68) |
| Methotrexate | 18 | (58) |
| Infliximab | 12 | (39) |
| Azathioprine | 8 | (26) |
| Cyclosporine | 3 | (10) |
| Mycophenolate Mofetil | 3 | (10) |
| Leflunomide | 2 | (6) |
| Certolizumab | 1 | (3) |
| Abatacept | 1 | (3) |
| Values represent number with percentage (%). | | |
